# Copy Number Variants Captured by the Array Comparative Genomic Hybridization in a Cohort of Patients Affected with Hereditary Colorectal Cancer in Sri Lanka: The First CNV Analysis Study of the Hereditary Colorectal Cancer in the Sri Lankan Population

**DOI:** 10.31557/APJCP.2021.22.6.1957

**Published:** 2021-06

**Authors:** Prabhavi Wijesiriwardhana, Kalum Wetthasinghe, Vajira H W Dissanayake

**Affiliations:** 1 *Department of Medical Laboratory Science, Faculty of Allied Health Sciences, University of Ruhuna, Sri Lanka. *; 2 *Human Genetics Unit, Faculty of Medicine, University of Colombo, Sri Lanka. *

**Keywords:** Hereditary non polyposis colorectal cancer, copy number variants, next generation sequencing

## Abstract

**Introduction:**

Hereditary non-polyposis colorectal cancer (HNPCC), is an autosomal dominant disorder characterized by the development of multiple cancer types. Molecular diagnosis of HNPCC requires the precise identification of pathogenic germline variants in DNA mismatch repair (MMR) genes. Next Generation Sequencing (NGS) is now the gold standard test in practice, to identify these variants. However, large genomic rearrangements (LGR) in cancer predisposing genes (CPGs) are missed by NGS. This may lead to underestimation of the frequency of the variants, misleading the genetic diagnosis and delaying intervention in high risk individuals. Hence this study was aimed at identifying the presence of large genomic alterations that could explain the missing heritable risk of colon cancer in affected patients with family history strongly suggestive of hereditary colorectal cancer in Sri Lanka.

**Methods:**

A cohort of six patients affected with hereditary colorectal cancer who tested negative for pathogenic variants in next generation sequencing studies was investigated using Sure Print G3 Human CGH 4x180K microarray platform. Agilent Genomic-Workbench-v7.0.4.0 software was used to identify the Copy Number Variants (CNV). Four healthy individuals (>55years) were used as controls. Annotations of the CNV regions which were observed were done using the database of Genomic Variants.

**Results:**

We identified 150 CNVs including regions of both genomic gains and losses in the patient cohort. There was no difference in the average number or the average genomic burden of CNVs identified in the patients versus the controls. CNVs were residing on the positions of 1q21.2, 2q37.3, 2p11.2-p11.1, 5q13.2, 6p12.3, 7q31.33, 7p14.1, 14q32.33, 15q11.1-11.2, 16p11.2, 22q11.22, 22q13.1 that were assessed by the array platform used in the study. CNVs in any of the well-known common CPG s or CNVs that reside on or in close proximity to genes corresponding to MMR pathway were not identified. We found several distinct pathways that have previously been identified as having a direct association with the progression of HNPCC.

**Conclusion:**

This study shows that CNVs are likely contributors to the colorectal cancer predisposition in a small but significant proportion of patients affected with hereditary colorectal cancer in this cohort. Further studies have to perform to get a better understanding on the contribution of CNVs to the cancer predisposition in this cohort of patients in the Sri Lankan population.

## Introduction

Colorectal cancer (CRC) defined as the cancerous growths in the colon, rectum and appendix is also referred to as colon cancer or large bowel cancer (Sameer, 2013). CRC is now the third most common malignant disease in both men and women in Asia (Wong et al., 2019). According to the latest data published by the National Cancer Control Programme (NCCP) in Sri Lanka, the incidence of CRC in Sri Lanka has increased from a WHO age-standardized rate of 2.9/100,000 in 2001 (95%-confidence interval [95%-CI]: 2.64–3.16) to 6.08/100,000 in 2010 (95%-CI: 5.71–6.44). This is an estimated annual percentage change (EAPC) of 8.9 (95%-CI: 7.5–10.4) (NCCP,2016 ). Such alarming figures highlights the importance of identifying individuals at-risk of cancer so that early detection, treatment and appropriate preventive measures could be instituted. 

It is estimated that 5-10% of all cancers are with a hereditary predisposition harboring germline variants in high, moderate and low risk cancer predisposing genes (CPGs) (Nagy and Sweet, 2004).). Hereditary non-polyposis colorectal cancer (HNPCC), accounts for approximately 5-8% of all CRC patients (Lynch and Chapelle, 1999). Genomic variants in MLH1, MSH2, MSH6 and PMS2 genes have been identified as having hereditary predisposition to patients affected in such families (Wong et al., 2019, Lynch and Chapelle, 1999). Identification of the individuals bearing germline mutations in CPGs having gene-specific risk for the development of hereditary cancers will determine the selection of a candidate for participation in highly targeted cancer surveillance and management programmes

The clinical diagnosis of HNPCC is defined by the Amsterdam criteria that enables the identification of the genetic basis of the disease (Vasen et al.,1991). Traditionally, a personal and/or family history of cancer combined with phenotypic clues have been used to guide the selection of genes that are most likely to have an underlying pathogenic variant. The most likely CPGs were then evaluated to identify them. Recently, next-generation sequencing (NGS) technologies have been used to identify the pathogenic germline variants in CPGs that could lead to the cancer predisposition in affected patients with strong family history of hereditary cancers. However, in the Sri Lankan context, a substantial fraction of patients affected with HNPCC, the genetic etiology for the disease remained unexplained. Taking effort to explain this missing heritability will be helpful in categorizing the spectrum of genomic variants in CPG in the Sri Lankan population which has not previously been described.

In other populations, Copy number variants (CNVs) are been used to explain the missing heritable risk of HNPCC (Kemp et al., 2004). Copy number variants represent a class of structural variants involving regions of gains or losses of genomic material that can encompass large stretches of genomic sequence ranging from megabases (Mb) to a few kilobases in size (kb) (Peltomaki,2001). When CNVs incorporate into coding and promotor regions of the CPGs, they could affect epigenetic regulations and leading to the disease development. CNVs have been implicated in the development of many forms of CRC, e.g., previous studies have established that the most frequent CNVs in CC are CN gains at chromosomes 7p, 7q, 13q, 20p, 20q, Xp and Xq and CN losses at chromosomes 8p, 17p, 18p and 18q.27 (Xie et al., 2001). All these studies have led to the identification of multiple oncogenes (EGFR, ERBB2, CCND1, MET, MYC) and/or tumor suppressors (TP53, APC, SMAD4) (Lin et al., 2011, Eldai et al., 2013). This evidence suggests that a proportion of HNPCC families may be accounted for by genomic rearrangements that may not be readily identified using more traditional gene mutation search. However there has been a notable lack of consistencies across these studies probably because of differences in sample populations and technical issues on the array methods or the software used for the analysis. We carried out a patient control analysis recruiting ten patients affected with hereditary breast cancer with a family history strongly suggestive of hereditary breast cancer and four controls derived from the same population to identify the CNVs which could have been the genetic cause for the hereditary cancer predisposition on them. This study represent the first CNV analysis study conducted in the Sri Lankan patients affected with hereditary breast cancer.

## Materials and Methods

*Ethical approval*

Ethical clearance was obtained from the Ethics Review Committee of the Faculty of Medicine, University of Colombo [EC-17-136]. All those undergoing genetic testing were offered comprehensive pre- and post-test counseling and written informed consent was obtained prior to testing. Demographic and clinical data including gender, age, personal and family cancer histories were obtained. 

*Sample*

With the view to implementing pre symptomatic genetic testing in Sri Lanka a research project was designed to understand the genetic variants found in CPGs in a Sri Lankan cohort of patients with hereditary cancer. Two hundred patients affected with inherited cancer syndromes were recruited for the study and they all were subjected to NGS testing using the Illumia TruSight Cancer® gene panel that tests 94 CPGs associated with both common (e.g. breast, ovarian, endometrial, colorectal, prostate, gastric, pancreatic and thyroid) and rare hereditary cancers. Sequencing was followed by analysis of the data using a bioinformatics pipeline. The preliminary results of this component has shown that some patients with hereditary breast cancer did not contain any variant in CPGs. This raised the question as to whether those patients who did not have variants harbor large structural variants in CPGs that could predispose to hereditary cancer syndromes and this study was developed to fulfill the gap aiming to identify the missing heritability predisposing hereditary cancers. Out of the cases who were identified as variant negative, were selected for the study developing the hypothesis that they might actually be having larger variants that could have been missed by the NGS testing. Identifying those cases is important both for the patient and at risk relatives, with clinical management implications both for affected and unaffected individuals. This study includes six such individuals affected with HNPCC. Genetic predisposition to cancer was made based on National Comprehensive Cancer Network (NCCN) criteria [https://www.nccn.org]. They were also negative for Multiplex ligation dependent Probe Amplification assay (MLPA) which was performed afterwards. 

*Control sample*

Four healthy individuals (age>55yrs) with no known family history of cancer were also selected as controls for the study. Genomic DNA was extracted from peripheral venous blood samples using QIAamp DNA Blood Mini Kits (Qiagen, Germany) according to the manufacturer’s protocol. DNA concentrations were determined by using the Qubit 3.0 fluorimeter (Life Technologies).

*Genotyping and data processing*

Array Comparative Genomic Hybridization (aCGH) was performed on oligonucleotide-based Sure Print G3 Human CGH 4x180K microarray platform, according the protocol provided by the manufacturer. In brief, 1 μg in final volume of 13 μL of normal female control DNA – reference DNA (DNA universal control-Promega Madison WI USA- Woman Reference: G152A) and patient’s DNA were differentially labeled with Cy3 (cyanine 3-deoxyuridine triphosphate) and Cy5 (cyanine 5-deoxyuridina triphosphate), respectively, using Agilent SureTag Complete DNA Labeling Kit (Agilent Technologies). Labeled DNA was then cleaned with purification columns (Agilent Technologies) and hybridized on array at 65°C for 24 hours, according to manufacturer’s recommendations. Microarrays were washed using Agilent Oligo aCGH Wash Buffers and scanning was performed using Agilent SureScan Microarray Scanner according to manufacturer’s instructions (Agilent Technologies).

CGH data were extracted from scanned images (TIFF files) using Feature Extraction software(v11.0.1.1). Feature Extraction was used to extract foreground signal for background subtraction and to correct dye biases for LOWESS normalization. It employs a process by which are extracted from the scanned microarray image and translated into log10 ratios of the Cy3(green) labeled normal DNA and Cy5(red) labeled tumor DNA signals in each of all probes, allowing us to measure DNA copy number changes in the experiments using Agilent Genomic Workbench v7.0.4.0 software.The raw data(FE file) of log10 ratios were transformed to log2 ratios within Agilent Genomic Workbench software. Aberrations were detected with the ADM-2 algorithm, whose threshold was 6.0, and aberration filtering options. We defined gains and losses over a continuous 3 probes and a linear log2 ratio average of >=0.25 or <= -0.25, respectively. CNVR (Copy Number Variation Region) was defined as the union of more than 90 percent overlapping aberrant segments across multiple samples. MCR (Minimal Common Region) was defined as 100 percent overlapping common region more than at least 2 individual segments in CNVR. There are several MCRs in CNVR according to possible overlapping frequency. We annotated the Common CNV region to observed CNVs/CNVR/MCR using DGV DB (Database of Genomic Variants, http://dgv.tcag.ca/dgv/docs/GRCh37_hg19_variants_2015-07-23.txt).To visualize the individual aberration pattern, we plotted the logR ratio plot according to chromosome for each sample. All statistical method and visualization of individual aberrant region were conducted using R statistical language v.3.3.3 (www.r-project.org).

To identify the genes involved in the CNVs further, we queried the UCSC database (http://genome.ucsc.edu), Ensemble (http://www.ensembl.org), and BioGPS, (http://biogps.gnf.org). Gene annotation and gene overlap were determined using the human genome build 19 (hg19) and several widely used online databases (Ensembl: http://www.ensembl.org; UCSC: http://genome.ucsc.edu; and NetAffx: http://www.affymetrix.com). In addition, we have searched published data on common CNVs and colorectal cancer risk to assess the possible contribution of this type of variations to hereditary colorectal cancer in this cohort.

## Results

*Array resolution and CNV detection*

Several characteristics of the CNVs that were identified in colorectal cancer affected cases were compared with the CNVs observed in the controls.

Analysis of array data revealed a total of 150 CNVs in 06 individuals assessed in this study (Table 02). CNVs detected ranged in size from 19.122 Kb to 2777.11 Kb. There was no difference in the average number of CNVs identified in the patients versus the controls (p = 0.629). The average genomic burden of CNVs also did not differ between patients (11225.276 Kb) and controls (9584.264 kb), p = 0.09; or the average CNV size between patients (411.182 Kb) and controls (372.204 Kb), p = 0.299 (Table 1). 

*Demographic data*

In this study we analyzed six patients affected with HNPCC who had been previously identified as no single nucleotide variants in various high, moderate low risk CPGs. Family histories and clinocopathological details were obtained using a predesigned questionnaire and were mentioned in Table 1. The mean age of onset of the cancer was 45.2 years, ranging from 32 – 57 years. All patients reported at least one case of HNPCC in the family diagnosed at age <55 years and reported more than one case of HNPCC in the family history. Meanwhile, two patients had a family historyof HNPCC only, and one patients with a family history of Familial Adenomatous Polyposis (FAP) while others had family history of HNPCC including other cancer types such as breast, ovary and colon.

*Occurrence and distribution of CNVs in the cohort*

**Figure 1:**
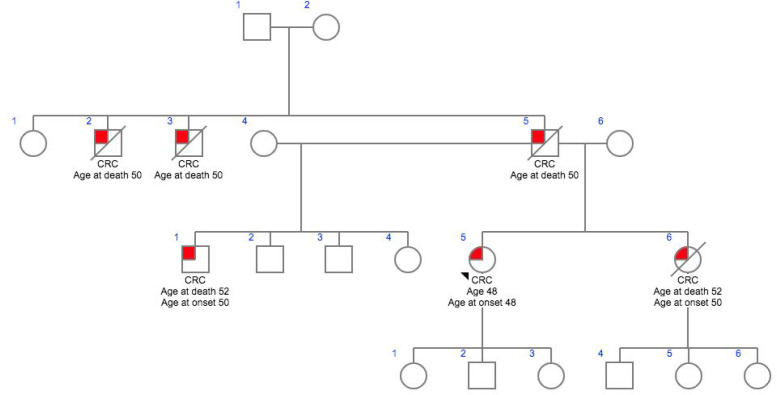
Family 1- CG007

The analysis revealed 150 and 103 CNVs in patients’ and control cohorts, respectively. Out of the identified CNVs 36.53% and 29.79% of CNV losses were seen in patients’ and control cohorts, respectively.75 CNVs were seen in both patients’ and control cohorts hence they were removed from further analysis. CNVs observed in both patient and control samples corresponded to 50.0% of the total, all of which overlapped common CNVs (DGV). The number of losses and gains in each chromosome in each patient was calculated separately (Figure 1). Highest number of CNVs were observed in chromosome 22 and 14. There were no CNVs found in chromosome 11, 18, 19, 20 and 21, respectively. Only one CNV was obtained in 02 chromosomes (chromosome 7 and 9). Out of the 75 CNVs unique to the patient cohort, 08 were shown to have common in two patients; three were common to three patients; and four were common to two patients, respectively (Table 3).

No CN gains or losses were identified within the defined search region for any of the 22 genes (*EXO1, LIG1, MLH1, MLH3, MSH2, MSH3, MSH6, PCNA, PMS1, PMS2, POLD1, POLD2, POLD3, POLD4, RFC1, RFC2, RFC3, RFC4, RFC5, RPA1, RPA2 and RPA3*) in the MMR pathway for all samples utilized in this study, patients and controls. 

Of the 75 CNVs identified unique to the patients, of these regions 56.5% of them have been previously reported in the Database of Genomic Variance (DGV, http://projects.tcag.ca/variation/). 

A total of 08 genomic regions were identified in two patients (06 gains and 02 losses); three common genomic regions were identified in three patients, located on chromosomes 10 and 14; and four CNV gains were identified in four patients on chromosome 1,2,14 and 22 (Table 03). Additional studies are required to investigate the sequence content of these regions to identify if novel contributors to disease development may reside in these regions.

From the 75 unique CNVs associated with genes identified in the patients, there were CNVs that affected the same gene even in multiple patients (Table 5). A total of 154 genes associated with 33 CNVs were identified in multiple individuals. Genes that were disrupted by the identified CNVs that were in multiple patients were seen; Eighty four genes as 2 CNV events; 23 genes as three CNV events and 32 genes as 4 CNV event.

*Gene set enrichment analysis*

Overrepresentation enrichment analysis of the CNVs was conducted using EnrichR, a bioinformatics web-based tool that contains a large collection of more than 100 gene set libraries (https://maayanlab.cloud/Enrichr/enrich#). These online data bases provides a comprehensive set of functional annotation tools for investigators to understand biological meaning behind large list of genes and to identify genomic loci associated with genetic disorders including cancer. The software was then used to compare the genes associated with CNVs uniquely identified across all patients (compared to controls) to all genes in the human genome. 

Studies have suggested that changes occurring in metabolic pathways are commonly observed during carcinogenesis (Hammoudi et al., 2011). In the absence of any cancer related pathway in the control population would suggest that those identified in the patient cohort are likely to be involved in some aspect of malignancy.

KEGG 2019 Human; analysis revealed a total of 07 significant pathways in which genes uniquely identified in the patients were enriched (Table 5). In the context of this study, these results suggest the potential existence of a germline predisposition in the affected patients which lead to metabolic conditions that promote disease development. WikiPathways 2019 Human; has identified 08 significant pathways having possibilities of the progression of colon cancer. The Peptide GPCRs pathway, Glutathione metabolism were also identified to be enriched and have been shown to play a role in gut permeability and motility (Bar-Shavit et al.,2016, Traverso et al., 2013). Eicosanoid Synthesis WP167 pathway, Selenium Micronutrient Network WP15 pathway, Imatinib and Chronic Myeloid Leukemia WP3640nthat were disrupted by the CNVs identified in this cohort have been well documented for their contribution to CRC (Wang and Dubois, 2010, Samei et al., 2016). Overall, our KEGG results suggest the importance of genetic risk factors which may act to promote the development of cancer.

**Figure 2:**
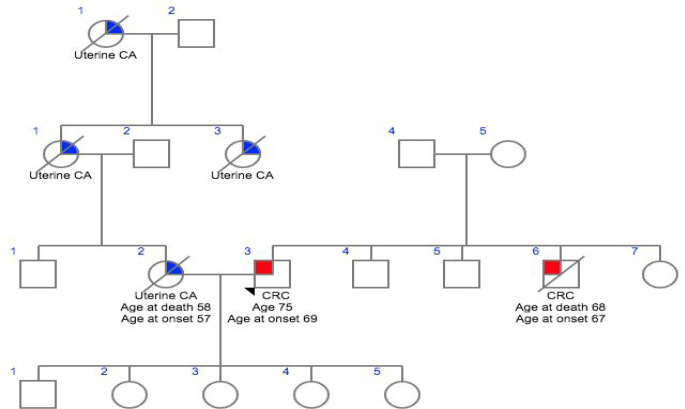
Family 2- CG016

**Figure 3:**
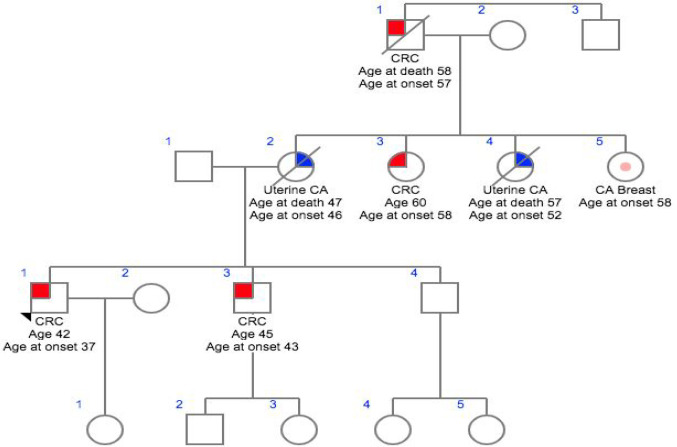
Family 3- CG018

**Figure 4:**
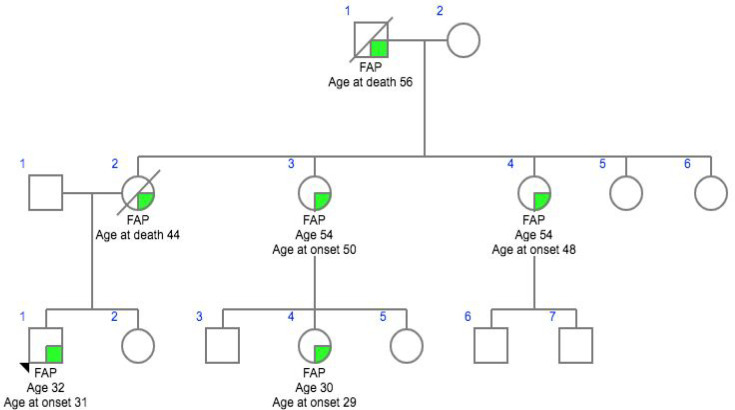
Family 4- CG033

**Figure 5:**
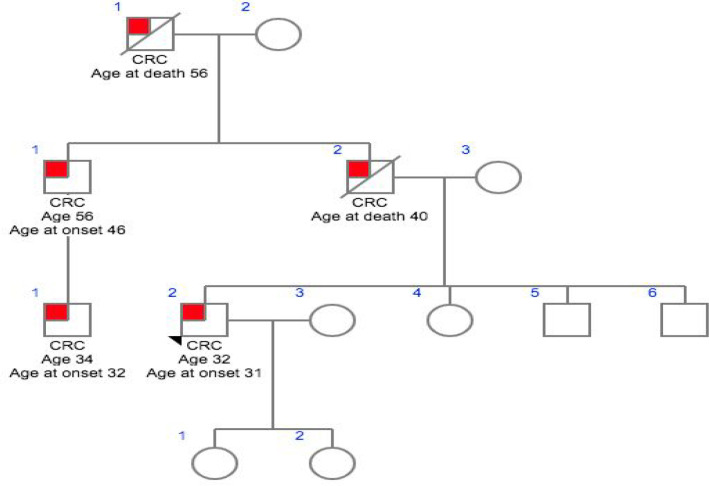
Family 5- CG036

**Figure 6.**
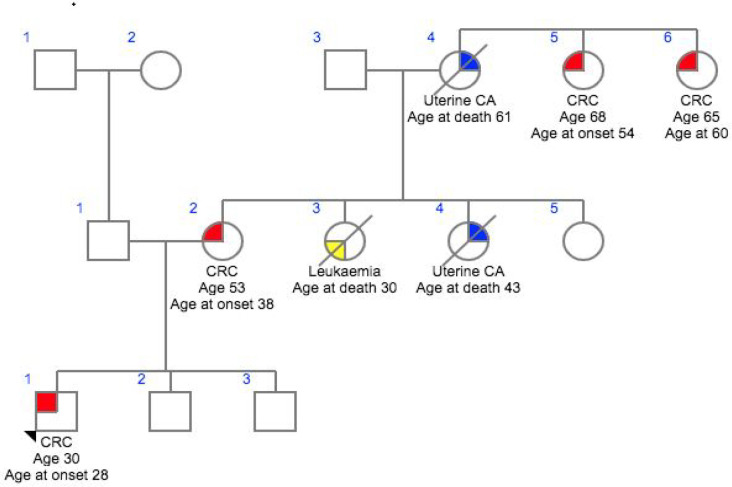
Family 6- CG239

**Table 1:** Summary of CNV Results of the CRC Affected Cases and Controls

	CNV number			CNV size (kb)		
	Total CNVs per group		Mean CNVs per sample	Total CNV affected genome per group	Mean total CNV affected genome per sample	Mean size of a CNV
Patients	6	150	25	67557.18	11260	324.223
Controls	4	104	26	38337.06	9584	352.352
p value			0.629		0.09	0.299

**Table 2:** Clinical Data and Family History of the Selected Cases

Family	Pt. ID	Age of onset	Sex	Cancer	Histology	Family	1^st^ degree	2^nd^ degree	3^rd^ degree
1	7	48	F	CRC	MDACA	Colorectal CA	Colorectal CA (2)	Colorectal CA (3)	-
2	16	57	M	CRC	MDACA	Breast, ovarian, Colorectal CA	Breast(2), Ovary (1)	Breast, Colorectal CA	Colorectal CA
3	18	37	M	CRC	MDACA	Uterine, colorectal, uterine, breast, thyroid	Uterine, Colorectal	Colorectal, Uterine, Breast	Colorectal
4	33	42	M	CRC	FAP	FAP	FAP	FAP	FAP
5	36	32	M	CRC	MDACA	Colorectal CA	Colorectal CA	Colorectal (2)	
6	239	57	M	CRC	MDACA	Colorectal CA ,Uterine CA, Leukaemia, Stomach CA	Colorectal CA	Leukaemia, Uterine (2)	Colorectal(2)

**Table 3:** Genomic regions associated with unique CNVs identified in multiple patients

Chr:	Cytoband	Start	End	Size (kb)	Patient
2 CNV Gains					
2	q37.3	242998314	243041364	43.05	GC07,239
5	q13.2	68849594	70369959	1520.36	CG16,18
22	q11.22	22458410	23238919	780.51	CG07,239
15	q11.1-q11.2	20432851	22558756	212.59	CG07,33
16	p11.2	32573808	33961233	1387.42	CG07,33
17	q12	34437475	34475514	38.04	CG 16,36
2 CNV losses					
15	q11.2	22304596	22558756	254.16	CG18,36
22	q13.1	39294332	39385485	91.15	CG36,239
3 CNV Losses					
10	q11.22	46158097	47702587	1544.5	CG16,18,33
10	q11.22	46976157	47702587	726.43	CG07,36,239
14	q32.33	106531557	106559103	27.55	CG16,33,239
4 CNV Gains					
1	q21.2	147824148	149378266	1554.12	CG16,18,36,239
2	p11.2-p11.1	89129532	91906643	2777.11	CG16,18,36,239
14	q32.33	106334907	106354441	19.53	CG16,18,33,239
22	q11.22	22458410	23245888	787.48	CG07,16,33,36

**Figure 6:**
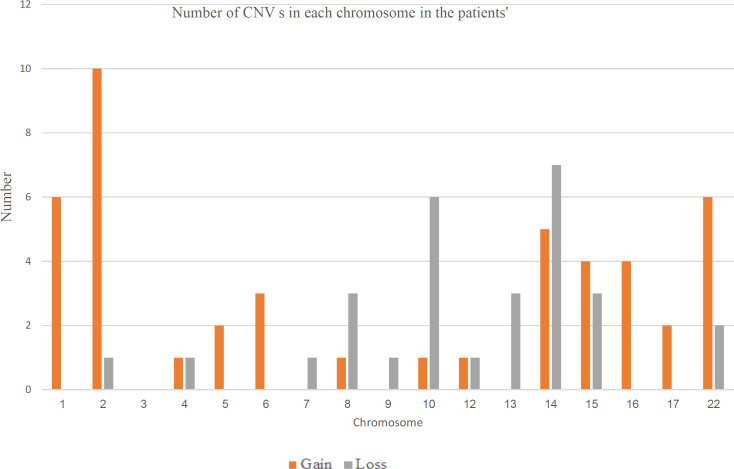
Overview of the Number of Gains and Losses in Each Chromosome

**Table 4:** Genes Associated with Unique CNVs (Compared to Controls) Identified Across Multiple Patients. Number of CNV events in which gene (s) have been identified with the CNV type

Type	2 Events	3 Events	4 Events
Gains	AJ004954,CHEK2P2,CT60,CXADRP2,DQ571479,DQ572979,DQ573684,DQ576041,DQ578838,DQ582073,DQ582260,DQ582939,DQ583164,DQ587539,DQ590589,DQ592463,DQ594309,DQ595048,DQ595648,DQ600342,DQ786202,GOLGA6L6,GOLGA8CP,HERC2P3,HERC2P7,JB175342,LOC646214,LOC727924,NBEAP1,NF1P2,OR4M2,OR4N3P,OR4N4,POTEB,REREP3,AK300387,BC041879,BC068290,DQ571479,DQ574674,IGH,JF934746,LINC00273,LOC390705,RNU676P,SLC6A10P,TP53TG3,TP53TG3B,X69637,CCL4,LOC728323,BCR,DKFZp667J0810,DQ570150,DQ575049,DQ597441,GGTLC2,IGLL5,LOC648691,LOC96610,MIR650,POM121L1P,PRAME,VPREB1,ZNF280A,ZNF280B,AK124130,AX748379,BC045789,DQ570150,DQ570835,DQ571461,DQ574682,DQ575504,DQ587763,DQ591060,DQ596042,GTF2H2,GTF2H2B,GTF2H2C,GUSBP3,GUSBP9,LOC100272216		AF380582,AK094156,AK310441,AK311108,BC023516,BC062745,DQ577785,DQ786323,FCGR1C,FLJ39739,LOC388692,LOC645166,NBPF14,NBPF15,NBPF16,NBPF8,NBPF9 AK128525,DQ571479,DQ576039,DQ576041,LOC654342, BCR,DKFZp667J0810,DQ570150,DQ575049,DQ597441,GGTLC2,IGLL5,LOC648691,LOC96610,MIR650,POM121L1P,PRAME,VPREB1,ZNF280A,ZNF280B
Loss	AJ004954,LOC727924,OR4M2,OR4N3P,OR4N4,REREP3,APOBEC3A,APOBEC3A_B,APOBEC3B	AGAP4,AGAP9,AK057316,AK309109,ANXA8,BMS1P1,BMS1P6,DQ577099,DQ588224,FAM21C,FAM25C,FAM35BP,FAM35DP,FRMPD2,GLUD1P7,GPRIN2,HNRNPA1P33,LINC00842,NPY4R,PTPN20A,PTPN20B,SYT15,ZFAND4,	

**Table 5:** Candidate CNVs That are Likely Associated with Malignancies

Index	Name	P-value	Adjusted p-value	Odds Ratio	Combined score	Genes
1	Staphylococcus aureus infection	0.001162	0.003487	46.43	313.75	ZNF705G, DEFB103A, DEFB104B SPAG11B, FAM66B, USP17L1P DEFB106A, DEFB107B, DEFB105A USP17L4, DEFB107A, FAM90A10P FAM90A7P, DEFB4B, DEFB109P1B
2	NOD-like receptor signaling pathway	0.007664	0.0115	17.32	84.35	ZNF705G, DEFB103A, DEFB104B SPAG11B, FAM66B, USP17L1P DEFB106A, DEFB107B, DEFB105A USP17L4, DEFB107A, FAM90A10P FAM90A7P, DEFB4B, DEFB109P1B
3	IL-17 signaling pathway	0.06755	0.06755	15.44	41.62	ZNF705G, DEFB103A, DEFB104B SPAG11B, FAM66B, USP17L1P DEFB106A, DEFB107B, DEFB105A USP17L4, DEFB107A, FAM90A10P FAM90A7P, DEFB4B, DEFB109P1B
4	Neuroactive ligand-receptor interaction	0.185	0.185	5.3	8.94	AK057316, AK309109, DQ588224, NPY4R, HNRNPA1P33, AGAP9, GPRIN2,FAM25C,ANXA8,FAM35DP, BMS1P6, LINC00842
5	Olfactory transduction	0.07826	0.07826	4.65	11.85	DQ571479,DQ590589,REREP3 OR4N4,OR4M2,NBEAP1,DQ583164 DQ582260,NF1P2, OR4N3P,AJ004954 CT60,DQ573684, LOC646214, POTEB DQ595048,LOC727924,DQ786202 CXADRP2, DQ587539,DQ576041
6	Chronic myeloid leukemia	0.05553	0.1111	18.96	54.82	LOC96610, DKFZP667J0810,IGLL5 BCR,GGTLC2,ZNF280A,VPREB1 MIR650,ZNF280B,DQ575049,PRAME DQ597441,POM121L1P,DQ570150 LOC648691
7	Pathways in cancer	0.3317	0.3317	2.63	2.9	LOC96610, DKFZP667J0810,IGLL5 BCR,GGTLC2,ZNF280A,VPREB1 MIR650,ZNF280B,DQ575049,PRAME DQ597441,POM121L1P,DQ570150 LOC648691
WikiPathways 2019 Human						
1	Peptide GPCRs WP24	0.04352	0.08704	24.8	77.74	AK057316,AK309109,DQ588224 NPY4R,HNRNPA1P33,AGAP9 GPRIN2, FAM25C,ANXA8,FAM35DP BMS1P6, LINC00842
2	GPCRs, Class A Rhodopsin-like WP455	0.1438	0.1438	7.01	13.59	AK057316,AK309109,DQ588224 NPY4R,HNRNPA1P33,AGAP9 GPRIN2, FAM25C,ANXA8,FAM35DP BMS1P6, LINC00842
3	Gamma-Glutamyl Cycle for the biosynthesis and degradation of glutathione, including diseases WP4518	0.007476	0.03512	158.54	776.21	LOC96610, DKFZP667J0810,IGLL5 BCR,GGTLC2,ZNF280A,VPREB1 MIR650,ZNF280B,DQ575049,PRAME DQ597441,POM121L1P,DQ570150 LOC648691
4	Imatinib and Chronic Myeloid Leukemia WP3640	0.0149	0.03512	75.06	315.73	LOC96610, DKFZP667J0810,IGLL5 BCR,GGTLC2,ZNF280A,VPREB1 MIR650,ZNF280B,DQ575049,PRAME DQ597441,POM121L1P,DQ570150 LOC648691
5	Selenium Micronutrient Network WP15	0.06262	0.08766	16.72	46.33	LOC96610, DKFZP667J0810,IGLL5 BCR,GGTLC2,ZNF280A,VPREB1 MIR650,ZNF280B,DQ575049,PRAME DQ597441,POM121L1P,DQ570150 LOC648691
6	NRF2 pathway WP2884	0.2144	0.2144	4.42	6.8	LOC96610, DKFZP667J0810,IGLL5 BCR,GGTLC2,ZNF280A,VPREB1 MIR650,ZNF280B,DQ575049,PRAME DQ597441,POM121L1P,DQ570150 LOC648691
7	Eicosanoid Synthesis WP167	0.02007	0.03512	54.83	214.32	LOC96610, DKFZP667J0810,IGLL5 BCR,GGTLC2,ZNF280A,VPREB1 MIR650,ZNF280B,DQ575049,PRAME DQ597441,POM121L1P,DQ570150 LOC648691
8	Glutathione metabolism WP100	0.01712	0.03512	64.81	263.64	LOC96610, DKFZP667J0810,IGLL5 BCR,GGTLC2,ZNF280A,VPREB1 MIR650,ZNF280B,DQ575049,PRAME DQ597441,POM121L1P,DQ570150 LOC648691

## Discussion

To our knowledge, this is the first CNV analysis of colorectal cancer, done on the Sri Lankan population. Compared to the studies on single nucleotide variants in colorectal cancer predisposing genes, the contribution of inherited copy number variation to colorectal cancer risk remains relatively understudied. Out of the cases who were identified as variant negative, were selected for the study developing the hypothesis that they might actually be having larger variants that could have been missed by the NGS testing. Identifying those cases is important both for the patient and at risk relatives, with clinical management implications both for affected and unaffected individuals.

In this study we provide evidence that CNVs are a potential explanation for some of the patients affected with hereditary colorectal cancer who do not harbour germline variants in known susceptibility genes. Samples were processed on one platform and analysed using the same analysis software and experimental parameters inorder to ensure uniqueness and to reduce biasness that could be occurred. The common guidelines for interpretation of the possible phenotypic impact of CNVs include comparing them with genomic imbalances recorded in healthy individuals.The Database of Genomic Variants (DGV) compiles 42 worldwide peer-reviewed studies on structural variations (deletions, duplications and inversions, mostly >1 Kb) in control samples. Comparison between the number and size of CNVs between patients and controls did not reveal any significant differences inbetween cohorts. Limited number of samples and controls utilized in the current study may represents a potential bias, but several other studies also have shown similar results to the current study.

Since we could not identified CNVs in any of the well-known common CPG s in these patients, we tried to find out the CNVs that reside on or in close proximity to genes corresponding to MMR pathway. We were unable to identify any DNA mismatch repair genes targeted by CNVs that may contribute to the genetic etiology of HNPCC patients recruited into this study. We have identified many CNVs that resides on various other oncogenes where we can get some evidence to prove the hypothesis we have. Also we identified several genomic regions that were altered in multiple unrelated HNPCC patients that could potentially be associated with disease risk. It is interesting to note that the CNVs overlapping genes have been implicated in biological processes involved in different pathways. 

Common CNVs often contain cancer-related genes and likely play a role in carcinogenesis. In our study, the numbers of unique CNVs per genome were quite high in patient’s cohort. Nevertheless, the patients did present a higher proportion of rare CNVs compared to controls. Assuming that some of these rare CNVs are cancer-related, the patients would carry an increased cancer risk proportionate to the number of rare genomic imbalances. However, the connection between this finding and the patients’ phenotypes needs to be investigated.

We performed pathway analysis aiming to identify possible common pathways associated with the heterogeneous outcomes of our analysis. We found several distinct pathways that have previously been identified as having a direct association with the progression of HNPCC. Results from WiKi 2019 pathway analysis identified the enrichment of pathways involved in metabolism, and these are known to be required for disease development. It is likely that these loci may contribute to CRC disease risk in the affected cases in the present study.

In conclusion, this study has revealed that there are a number of CNVs which may contribute to the hereditary predisposition of HNPCC. We propose that variants in these rare genes may account for disease in a significant proportion of patients affected with HNPCC in Sri Lanka. Overall the results of this study provide further grounds for further investigation into the presence of CNVs in larger series of patients who do not harbor changes in known colorectal cancer susceptibility genes.

## Author Contribution Statement

PW conceived the study, carried out the molecular genetic studies, acquired the data and interpretation of the data, carried out the statistical analysis and drafted the manuscript. VHWD recruited the cases to the Genetic Clinic at the Human Genetics Unit, Faculty of Medicine, University of Colombo, Sri Lanka and examined them. KW and VHWD were supervised the study. All authors read and approved the final version of the manuscript to be published.
